# PARENTAL INFLUENCE AND MULTIPLE NICOTINE PRODUCT USE PATTERNS AMONG ADOLESCENTS: A CROSS-SECTIONAL STUDY OF FAMILY CONTEXT AND E-CIGARETTE USE

**DOI:** 10.13075/ijomeh.1896.02618

**Published:** 2025

**Authors:** Karolina Zajdel, Anna Merecz-Sadowska, Arkadiusz Sadowski, Aneta Jęcek, Magdalena Bakalarz, Dorota Kaleta

**Affiliations:** 1 Medical University of Łódź, Department of Medical Informatics and Statistics, Łódź, Poland; 2 University of Łódź, Department of Economic and Medical Informatics, Łódź, Poland; 3 Medical University of Łódź, Department of Allergology and Respiratory Rehabilitation, Łódź, Poland; 4 Medical University of Łódź, Department of Hygiene and Epidemiology, Łódź, Poland

**Keywords:** risk perception, nicotine dependence, adolescent health, family influence, e-cigarette use, multiple nicotine product use

## Abstract

**Objectives:**

Adolescent e-cigarette use is a growing public health concern, yet the influence of family context and risk perceptions on usage patterns remains poorly understood.

**Material and Methods:**

This cross-sectional study investigated relationships between family context, risk perceptions, and e-cigarette use patterns among a nationally representative sample of 4797 Polish adolescents aged 15–18 years who were current e-cigarette users (defined as use in the past 30 days). Using computer-assisted web interviews, the authors assessed family factors (parental awareness, attitudes, and nicotine use), risk perceptions, and e-cigarette use behaviors.

**Results:**

Among adolescent e-cigarette users, 92.6% engaged in poly-nicotine use (i.e., used ≥1 other nicotine product) with only 7.4% reporting exclusive e-cigarette use. Notably, 46.7% of participants reported extensive multiple product use (concurrent use of ≥5 products). Structural equation modeling demonstrated that family context significantly influenced e-cigarette use patterns, both directly (β = 0.31, p < 0.001) and indirectly through risk perceptions (β = 0.12). Risk perception emerged as the strongest direct predictor of e-cigarette use patterns (β = 0.41, p < 0.001). Parental e-cigarette use was associated with a 70% increase in adolescents' likelihood of intensive e-cigarette use (≥10 times daily) (OR = 1.70, 95% CI: 1.39–2.07, p < 0.001). Traditional cigarette initiation (compared to e-cigarette initiation) was associated with almost twice the likelihood of multiple nicotine product use (OR = 1.89, 95% CI: 1.67–2.13, p < 0.001).

**Conclusions:**

These findings highlight the significant influence of family context and risk perceptions on adolescent nicotine use behaviors, suggesting that family-based interventions addressing these factors could be effective prevention strategies.

## Highlights

- Among adolescent e-cigarette users, 92.6% engage in multiple product use.
- Family context influences use both directly and indirectly.
- Risk perception is the strongest predictor of use patterns.
- Parental e-cigarette use increases adolescents' intensive use by 70%.
- Family influence is stronger in younger adolescents.

## INTRODUCTION

Adolescent e-cigarette use has become a significant global public health concern, with rates rising dramatically in many countries over the past decade. While e-cigarettes are sometimes marketed as less harmful alternatives to traditional cigarettes, mounting evidence indicates they pose considerable health risks to young people, including nicotine addiction and potential long-term respiratory and cardiovascular effects [1]. This trend is particularly evident in Poland, where recent data from the 2022 Global Youth Tobacco Survey (GYTS) indicate that 22.3% of adolescents reported current use of electronic cigarettes [2]. Moreover, adolescent e-cigarette use is frequently associated with the concurrent use of other nicotine and tobacco products – a pattern known as “poly-tobacco” use – which may amplify these health risks [3]. This phenomenon of multiple nicotine product use has been observed across diverse geographical regions, from North America and Western Europe to Asia and the Pacific [4,5].

Understanding the factors driving adolescent e-cigarette use, particularly multiple nicotine product use, is critical for developing effective prevention strategies. Individual factors, such as risk perception and sensation-seeking, and broader social influences, including peer pressure and social media exposure, contribute significantly [6]. However, the family environment remains a cornerstone of adolescent development and a powerful influence on substance use behaviors [7,8]. Parental smoking, whether traditional cigarettes or e-cigarettes, has consistently been linked to a higher likelihood of adolescent e-cigarette initiation and continued use [9,10]. Several mechanisms may explain this link, including social learning theory (where adolescents model parental behaviors), increased accessibility of nicotine products within the home, and shared genetic vulnerabilities to nicotine dependence [11,12].

Beyond parental smoking behavior, parental attitudes and awareness regarding e-cigarette use are also crucial. Parents who are unaware of their child's e-cigarette use cannot effectively intervene, while those with permissive attitudes towards e-cigarettes may inadvertently convey that such behavior is acceptable [13,14]. Conversely, strong parental disapproval of e-cigarettes, combined with open communication about the risks, can act as a protective factor [15,16].

This study aims to contribute to a broader understanding of family influences on adolescent e-cigarette and multiple nicotine product use by investigating these relationships within a Polish adolescent population. The authors expect to find that parental e-cigarette use is positively associated with both adolescent e-cigarette use and the use of multiple nicotine products. Furthermore, it is anticipated that lower parental awareness of their child's e-cigarette use will be linked to a greater likelihood of adolescent e-cigarette use and multiple product use. Similarly, the authors hypothesize that parental attitudes that do not actively oppose e-cigarette use will be associated with higher rates of adolescent e-cigarette and multiple nicotine product use. Finally, based on the hierarchical progression often observed in substance use, the authors predict that adolescents who initiate nicotine use with traditional cigarettes will be more likely to subsequently engage in multiple nicotine product use. The findings may enhance our understanding of family influence on adolescent health behaviors.

## MATERIAL AND METHODS

### Study design and setting

This study was designed as a nationwide, cross-sectional survey and was conducted as a key component of the Polish National Health Program (2016–2020), under the task entitled “Program for combating the health consequences of using tobacco and related products.” The primary objective was to gather data from a representative sample of adolescents across all of Poland. Using a computer-assisted web interview (CAWI) methodology, the data for this study were collected in a single wave in 2019 by DSC – Studio Cati Project on the SURneo platform. Subsequent statistical analysis and manuscript preparation followed. The sampling frame targeted students aged 15–18 years in upper secondary schools across all 16 Polish voivodships, ensuring national representativeness.

### Participants and sampling

Participants for this nationwide study were recruited based on specific inclusion criteria. To be eligible, an individual had to be:

– an adolescent aged 15–18 years,
– enrolled in an upper secondary school in Poland (general, technical, or vocational),
– a current e-cigarette user, defined as having used an e-cigarette at least once in the past 30 days.

Consequently, the study's findings are based exclusively on data from current adolescent e-cigarette users.

To recruit a nationally representative sample of these users, a multi-stage, stratified-random sampling design was employed. The sampling frame was stratified across all 16 Polish voivodships, as well as by school type, urbanization level, sex, and age to ensure the findings could be reliably generalized. An initial target of 200 schools was set, with replacement procedures implemented for non-responding schools.

The final sample comprised 4797 adolescents who met the inclusion criteria. The demographic breakdown of the sample was as follows: 57.8% males and 42.2% females; age distribution was 12.7% for 15-year-olds, 29.5% for 16-year-olds, 31.2% for 17-year-olds, and 26.6% for 18-year-olds. Regarding school type, 39.4% attended general high schools, while 60.6% attended technical or vocational schools. The sample was distributed between rural (42.9%) and urban (57.1%) areas of residence. The initial school-level response rate was 5.5%.

### Data collection procedures

The data collection process involved a 2-step procedure. First, upper secondary schools were recruited via a dedicated call center. School directors were provided with comprehensive information about the study's aims and its anonymous nature. Upon receiving consent from the school's administration, a unique, anonymous link to the online questionnaire was provided to a designated school representative (e.g., the director or a teacher).

In the second step, the school representative was responsible for distributing this link to eligible students. The survey was designed to be completed independently by the students on their personal devices (e.g., computers or smartphones) at a time of their convenience, likely outside structured class hours. The survey was self-administered and took approx. 10 min to complete. All participants were assured of complete anonymity, and no personally identifiable information was collected. The SURneo platform incorporated forced-choice responses, skip logic, and consistency checks to minimize missing data. A pilot test was conducted prior to main data collection, with necessary adjustments made based on feedback.

### Measures

The survey instrument gathered data on demographics, e-cigarette use patterns, multiple product use, family context, and risk perceptions. While the questionnaire was developed specifically for the Polish National Health Program, key items were adapted from established public health surveillance tools, such as the GYTS, and validated dependence scales to ensure construct validity.

Demographic data included age, gender, school type (general high school vs. vocational/technical), and residential setting (urban vs. rural).

E-cigarette use patterns were assessed using 3 key indicators: daily use frequency (once, 2–4 times, 5–9 times, 10–15 times, and >15 times/day), time to first use after waking – a core indicator of nicotine dependence adapted from the *Fagerström Test for Nicotine Dependence* (FTND) (≤5 min, 6–15 min, 16–30 min, 31–60 min and >60 min), and nicotine concentration in e-liquids (0 mg/ml, 1–3 mg/ml, 4–10 mg/ml, 11–19 mg/ml, and ≥20 mg/ml).

To assess concurrent multiple product use, participants, who were all defined as current e-cigarette users, were also asked about their past 30-day consumption of 8 other nicotine products, with use status recorded for each. The full list of assessed products included e-cigarettes (as the primary product), combustible tobacco (conventional cigarettes, hand-rolled cigarettes, cigars/cigarillos, and water pipes/shisha), heated tobacco products (HTPs), pod-based systems, and oral/smokeless products (snuff and snus).

Family context was assessed through several items. Parental awareness and attitudes toward the adolescent's e-cigarette use were assessed with a single item offering 4 response options: “they don't know i use them” (unaware), “they are aware, but have no specific opinion,” “they are supportive of my use,” or “they are opposed to my use.” Parental tobacco and nicotine use was measured separately for mothers and fathers across 2 product types including conventional cigarettes, and e-cigarettes, with a question about whether: “neither parent uses,” “only my mother uses,” “only my father uses,” or “both parents use.”

Finally, risk perceptions were measured by asking participants to compare the harmfulness of e-cigarettes to traditional cigarettes: “Compared to smoking traditional cigarettes, do you think using e-cigarettes is...?” Response options were: “much less harmful,” “somewhat less harmful,” “equally harmful,” “somewhat more harmful,” “much more harmful,” with an additional “I don't know” option. Primary motivation for use was determined through a single-choice question asking participants to identify their main reason for using e-cigarettes from a predefined list that included “enjoyment,” “smoking cessation aid,” “social reasons,” “addiction,” “other reasons,” and “uncertain.”

### Statistical analysis

Data analysis was performed using Statistica software v. 13.1. Descriptive statistics were calculated for all variables. Chi-square tests examined bivariate associations between demographic characteristics, e-cigarette use patterns, multiple product use, and family context variables. Multivariate logistic regression models were constructed to identify factors associated with 2 key dichotomous outcomes:

– intensive e-cigarette use, defined as using e-cigarettes ≥10 times/day,
– extensive multiple nicotine product use, defined as the concurrent use of ≥5 different nicotine product types.

Odds ratios (ORs) and 95% confidence intervals (CIs) were calculated for these models.

Structural equation modeling (SEM) using the lavaan package in R examined direct and indirect pathways between key constructs. The model specified 3 latent variables:

– family context – indicated by parental traditional cigarette use, parental attitudes, parental e-cigarette use, and parental awareness;
– risk perception – indicated by adolescents' perceived harmfulness of e-cigarettes relative to conventional cigarettes;
– e-cigarette use patterns – indicated by frequency of e-cigarette use, time to first use after waking, multiple product use, and nicotine concentration.

Model fit was assessed using multiple indices: comparative fit index (CFI), Tucker-Lewis index (TLI), root mean square error of approximation (RMSEA), and standardized root mean square residual (SRMR). Interaction effects between key variables were examined using interaction terms in regression models, and stratified analyses. Statistical significance was set at p < 0.05 for all analyses.

### Ethical considerations

This study was conducted under the ethical framework of the Polish National Health Program, supervised by the National Institute of Public Health – National Institute of Hygiene (Narodowy Instytut Zdrowia Publicznego – Państwowy Zakład Higieny), and adhered to the principles of the Declaration of Helsinki. A multi-level consent process was applied. First, institutional consent was obtained from school principals. Second, participants provided electronic informed assent after being presented with an information sheet detailing the study's voluntary and anonymous nature. Due to the completely anonymous, minimal-risk design, a waiver of active parental consent was utilized, a standard practice for such public health surveys. Participants were informed of their right to withdraw at any time by closing the survey.

## RESULTS

### Demographic characteristics

The study included 4797 adolescents aged 15–18 years who reported e-cigarette use within the past 30 days. Males constituted the majority (57.8%, N = 2775) of participants, with females representing 42.2% (N = 2022). The age distribution showed that most participants were 16–17 years old (60.7%, N = 2912), with 16-year-olds comprising 29.5% (N = 1414), 17-year-olds 31.2% (N = 1498), 18-year-olds 26.6% (N = 1277), and 15-year-olds – 12.7% (N = 608). The male-to-female ratio was highest among 18-year-olds (1.68:1) and lowest among 15-year-olds (1.10:1), with a statistically significant gender distribution across age groups (χ^2^ test, p < 0.001) showing increasing proportion of males with age.

Regarding educational setting, 39.4% (N = 1891) of participants attended general high schools, while 60.6% (N = 2906) attended technical or vocational schools. For residential distribution, 42.9% (N = 2056) lived in rural areas, while 57.1% (N = 2741) resided in urban areas of varying sizes: 21.6% (N = 1038) in small towns (<20 000 inhabitants), 19.5% (N = 935) in medium-sized towns (20 000–99 999 inhabitants), 11.6% (N = 556) in large cities (100 000–500 000 inhabitants), and 4.4% (N = 212) in metropolitan areas (>500 000 inhabitants). Significant residential variation in gender distribution was observed (p < 0.001), with rural areas showing the highest proportion of males (60.3% male vs. 39.7% female) and the largest cities showing a reversed pattern with female predominance (38.7% male vs. 61.3% female), suggesting that urbanization may influence gender-specific patterns of e-cigarette use.

### E-cigarette use patterns

Analysis of e-cigarette use patterns revealed significant variations in frequency, intensity, and temporal distribution. Regarding daily use frequency, while 46.2% of participants reported low frequency (1–4 times daily), a concerning 33.6% (N = 1611) reported high-frequency use (≥10 times daily). This included 21.1% (N = 1011) reporting 10–15 uses and 12.5% (N = 600) exceeding 15 uses/day, with moderate use (5–9 daily uses) reported by 20.2% (N = 968) of participants. Significant gender differences were observed (χ^2^ = 42.38, p < 0.001), with males more likely than females to report high-frequency use (36.8% vs. 29.0%). Age was also significantly associated with use frequency (χ^2^ = 63.17, p < 0.001), with high-frequency use increasing from 25.3% among 15-year-olds to 39.5% among 18-year-olds.

Time to first use after waking – a key indicator of nicotine dependence – showed concerning patterns, with 27.8% (N = 1333) reporting usage within 5 min of waking and an additional 27.0% (N = 1296) within 6–30 min of waking. This suggests potential nicotine dependence in more than half (54.8%) of the sample. Males were more likely than females to use e-cigarettes within 5 min of waking (30.9% vs. 23.5%, χ^2^ = 35.71, p < 0.001), with this early use pattern increasing with age from 20.4% among 15-year-olds to 32.7% among 18-year-olds (χ^2^ = 47.53, p < 0.001).

For nicotine liquid concentration, the majority (64.6%, N = 3,093) reported moderate to high concentrations (4–19 mg/ml), with males more likely to use high concentrations (≥11 mg/ml): 38.9% vs. 31.4% for females (χ^2^ = 31.26, p < 0.001). Vocational/technical school students reported higher nicotine concentrations compared to general high school students.

The combined analysis of these patterns indicates concerning levels of intensive e-cigarette use and potential nicotine dependence among a substantial proportion of adolescent users, with significant gender differences and age gradients suggesting progressive development of dependency characteristics throughout adolescence.

### Multiple nicotine product use patterns

Analysis of multiple nicotine product use revealed that only 7.4% (N = 357) of participants reported exclusive e-cigarette use, while the overwhelming majority (92.6%, N = 4440) engaged in poly-nicotine use, consuming ≥1 other nicotine product alongside e-cigarettes (Table 1). Within this group, a significant subset demonstrated high-intensity consumption patterns; notably, 46.7% (N = 2239) reported using ≥5 distinct nicotine products concurrently. This specific behavior, which the authors defined as “extensive multiple product use,” was treated as a key outcome variable in the logistic regression analysis.

**Table 1.** Patterns of multiple nicotine product use among adolescents by gender – a cross-sectional study among Polish adolescents aged 15–18 years, Poland, 2016–2020

Variable	Particiapnts (N = 4797) [n (%)]			p
	total	males (N = 2773)	females (N = 2024)	
Product type				
conventional cigarettes	4272 (89.1)	2503 (90.2)	1769 (87.6)	0.003
hand-rolled cigarettes	2876 (60.0)	1815 (65.4)	1061 (52.5)	<0.001
water pipe (shisha)	1851 (38.6)	1107 (39.9)	744 (36.8)	0.029
heated tobacco products	1508 (31.4)	902 (32.5)	606 (30.0)	0.055
pod-based system	1370 (28.6)	813 (29.3)	557 (27.5)	0.152
cigars/cigarillos	2172 (45.3)	1434 (51.7)	738 (36.6)	<0.001
snuff	2462 (51.3)	1582 (57.0)	880 (43.6)	<0.001
snus	299 (6.2)	207 (7.5)	92 (4.5)	<0.001
Number of products used				<0.001
e-cigarettes only	357 (7.4)	165 (5.9)	192 (9.5)	
2 products	729 (15.2)	384 (13.8)	345 (17.1)	
3 products	774 (16.1)	428 (15.4)	346 (17.1)	
4 products	698 (14.6)	385 (13.9)	313 (15.5)	
5 products	583 (12.2)	351 (12.6)	232 (11.5)	
6 products	580 (12.1)	358 (12.9)	222 (11.0)	
7 products	521 (10.9)	342 (12.3)	179 (8.9)	
8 products	359 (7.5)	246 (8.9)	113 (5.6)	
9 products	196 (4.1)	116 (4.2)	80 (4.0)	
Product initiation sequence				0.001
e-cigarettes first	1665 (34.7)	909 (32.8)	756 (37.4)	
conventional cigarettes	2834 (59.1)	1695 (61.1)	1139 (56.3)	
heated tobacco products	108 (2.3)	67 (2.4)	41 (2.0)	
snus	6 (0.1)	4 (0.1)	2 (0.1)	
snuff	184 (3.8)	100 (3.6)	84 (4.2)	

Significant gender differences were observed (χ^2^ = 54.87, p < 0.001), with males more likely to use a higher number of products concurrently (38.1% using ≥5 products vs. 29.8% of females). Age was also significantly associated with multiple product use (χ^2^ = 42.36, p = 0.012), with older adolescents engaging in more extensive polytobacco use.

Conventional cigarettes were the most commonly used product (89.1%, N = 4272), followed by hand-rolled cigarettes (60.0%, N = 2876), snuff (51.3%, N = 2462), and cigars/cigarillos (45.3%, N = 2172). Males showed significantly higher rates of use for hand-rolled cigarettes, cigars/cigarillos, and snuff (all p < 0.001).

Product initiation sequence analysis revealed that conventional cigarettes were the most common initial tobacco product (59.1%, N = 2834), followed by e-cigarettes (34.7%, N = 1665). Participants who initiated nicotine use with conventional cigarettes showed a higher probability of current multiple product use compared to those who initiated with e-cigarettes (χ^2^ = 147.23, p < 0.001). Among those who initiated with conventional cigarettes, 39.3% reported using ≥5 nicotine products currently, compared to 25.6% of those who initiated with e-cigarettes.

This extensive multiple product use raises significant public health concerns, as it may lead to increased nicotine exposure, stronger dependence, more difficult cessation, and potentially greater cumulative health risks compared to single product use.

### Family context analysis

Examination of family context revealed complex patterns of parental awareness, attitudes, and behaviors regarding adolescent e-cigarette use (Table 2). Analysis of parental awareness demonstrated that 37.5% (N = 1797) of parents were unaware of their adolescent's e-cigarette use. Among parents who were aware, reactions varied considerably: 23.0% (N = 1103) expressed no specific opinion, 34.2% (N = 1640) actively opposed their child's e-cigarette use, while only 5.4% (N = 257) were supportive.

**Table 2.** Family context and e-cigarette use among adolescents – a cross-sectional study among Polish adolescents aged 15–18 years, Poland, 2016–2020

Variable	Particiapnts (N = 4797) [n (%)]		p
	total	high-frequency e-cigarette use (≥10 times/day)	
Parental awareness and attitudes			<0.001
unaware	1797 (37.5)	626 (34.8)	
no specific opinion	1103 (23.0)	387 (35.1)	
opposed to use	1640 (34.2)	471 (28.7)	
supportive of use	257 (5.4)	127 (49.4)	
Parental e-cigarette			<0.001
neither parent	4356 (90.8)	1417 (32.5)	
mother only	125 (2.6)	51 (40.8)	
father only	222 (4.6)	94 (42.3)	
both parents	94 (2.0)	49 (52.1)	
Parental traditional cigarette use			<0.001
neither parent	2362 (49.2)	609 (25.8)	
mother only	582 (12.1)	218 (37.5)	
father only	963 (20.1)	406 (42.2)	
both parents	890 (18.6)	378 (42.5)	

Chi-square analysis revealed significant associations between parental awareness and adolescents' e-cigarette use intensity (χ^2^ = 38.62, p < 0.001). Among adolescents whose parents were supportive of e-cigarette use, 49.4% (N = 127) reported high-frequency use (≥10 times/day), compared to only 28.7% (N = 471) of those whose parents opposed such use.

Analysis of parental nicotine use patterns revealed distinct differences between traditional cigarette and e-cigarette use. For traditional cigarettes, 50.8% (N = 2435) of participants reported ≥1 parent who smoked: 20.1% (N = 963) reported father-only smoking, 12.1% (N = 582) mother-only smoking, and 18.6% (N = 890) reported both parents smoking. In contrast, parental e-cigarette use was considerably lower, with only 9.2% (N = 441) of participants reporting any parental e-cigarette use.

Significant associations were found between parental smoking status and adolescent e-cigarette use intensity (χ^2^ = 156.42, p < 0.001). Among adolescents with both parents smoking traditional cigarettes, 42.5% (N = 378) reported high-frequency e-cigarette use, compared to only 25.8% (N = 609) of those with non-smoking parents. Similarly, parental e-cigarette use showed strong associations with adolescent use patterns (χ^2^ = 41.73, p < 0.001), with 52.1% (N = 49) of adolescents reporting high-frequency use when both parents used e-cigarettes, compared to 32.5% (N = 1417) when neither parent used these products.

This comprehensive examination of family context suggests that parental awareness, attitudes, and smoking behaviors play significant roles in adolescent e-cigarette use patterns and use behaviors, highlighting the potential importance of family-based interventions.

### Risk perceptions and motivations

Analysis of adolescents' perceptions and motivations regarding e-cigarette use revealed important insights into the cognitive factors underlying nicotine use behaviors. The majority of participants (64.7%, N = 3104) perceived e-cigarettes as less harmful than traditional cigarettes, with 35.2% (N = 1688) viewing them as significantly less harmful and 29.5% (N = 1416) as somewhat less harmful (Table 3). A smaller proportion (16.5%, N = 793) considered e-cigarettes equally harmful to traditional cigarettes, while 9.2% (N = 441) perceived e-cigarettes as more harmful. Notably, 9.6% (N = 459) reported uncertainty about relative harm.

**Table 3.** Risk perceptions, motivations for e-cigarette use, and their relationship with high-frequency use – a cross-sectional study, among Polish adolescents aged 15–18 years, Poland, 2016–2020

Variable	Particiapnts (N = 4797) [n (%)]				p
	total	males (N = 2773)	females (N = 2024)	high-frequency e-cigarette use (≥10 times/day)	
Perceived harm compared to traditional cigarettes					<0.001
significantly more harmful	115 (2.4)	52 (1.9)	63 (3.1)	26 (22.6)	
more harmful	326 (6.8)	166 (6.0)	160 (7.9)	82 (25.2)	
equally harmful	793 (16.5)	408 (14.7)	385 (19.0)	198 (25.0)	
less harmful	1416 (29.5)	795 (28.6)	621 (30.7)	427 (30.2)	
significantly less harmful	1688 (35.2)	1093 (39.4)	595 (29.4)	702 (41.6)	
uncertain	459 (9.6)	261 (9.4)	198 (9.8)	176 (38.3)	
Primary motivation for use					<0.001
enjoyment	2430 (50.7)	1482 (53.4)	948 (46.9)	789 (32.5)	
smoking cessation aid	793 (16.5)	469 (16.9)	324 (16.0)	360 (45.4)	
social reasons	443 (9.2)	208 (7.5)	235 (11.6)	98 (22.1)	
addiction	144 (3.0)	89 (3.2)	55 (2.7)	82 (56.9)	
uncertain	459 (9.6)	249 (9.0)	210 (10.4)	143 (31.2)	
other reasons	528 (11.0)	278 (10.0)	250 (12.4)	139 (26.3)	

Significant gender differences were observed in risk perceptions (χ^2^ = 43.26, p < 0.001), with females more likely to perceive e-cigarettes as equally or more harmful than traditional cigarettes (29.8% vs. 22.6% among males). Age-related differences were also identified (χ^2^ = 38.72, p = 0.001), with older adolescents showing more balanced risk assessments.

Examination of motivations revealed that personal enjoyment was the predominant reason for e-cigarette use (50.7%, N = 2430), substantially outweighing smoking cessation (16.5%, N = 793) and social reasons (9.2%, N = 443). Only 3.0% (N = 144) explicitly acknowledged addiction as their primary motivation. Males were more likely to cite enjoyment (53.4% vs. 46.9% of females), while females more frequently reported social reasons (11.6% vs. 7.5%) (χ^2^ = 51.87, p < 0.001).

The relationship between risk perceptions and e-cigarette use intensity was significant. Participants who perceived e-cigarettes as significantly less harmful than traditional cigarettes reported the highest rates of intensive use (41.6%, N = 702), compared to only 22.6% (N = 26) among those who viewed them as significantly more harmful (χ^2^ = 129.35, p < 0.001). Similarly, those perceiving e-cigarettes as less harmful showed higher rates (30.2% and 41.6%) than those perceiving them as equally harmful (25.0%) or more harmful (25.2% and 22.6%). Participants with uncertain risk perceptions also demonstrated relatively high rates of intensive use (38.3%).

Primary motivation for use was strongly associated with usage patterns. Participants citing addiction as their primary motivation reported the highest rates of high-frequency use (56.9%), followed by those citing smoking cessation (45.4%). Those reporting social reasons showed the lowest rates of intensive use (22.1%), with enjoyment (32.5%) and other motivations (26.3% to 31.2%) falling in between (χ^2^ = 92.34, p < 0.001).

These findings suggest complex relationships between risk perceptions, motivations, and usage patterns, highlighting the importance of addressing these cognitive factors in developing effective intervention strategies.

### Multivariate analysis of nicotine use patterns

Multivariate logistic regression analysis revealed several significant predictors for both multiple nicotine product use (defined as using ≥5 products) and intensive e-cigarette use (defined as ≥10 times/day) among adolescents (Table 4).

**Table 4.** Multivariate logistic regression analysis of factors associated with multiple nicotine product use and intensive e-cigarette use – a cross-sectional study among 4797 Polish adolescents aged 15–18 years, Poland, 2016–2020

Factor	Multiple product use (≥5 products)			Intensive e-cigarette use (≥10 times/day)		
	OR	95% CI	p	OR	95% CI	p
Gender						
female (ref.)	1.00	–	–	1.00	–	–
male	1.46	1.30–1.64	<0.001	1.37	1.21–1.56	<0.001
Age (per year increase)	1.18	1.11–1.25	<0.001	1.22	1.15–1.30	<0.001
School type						
general high school (ref.)	1.00	–	–	1.00	–	–
vocational/technical	1.32	1.17–1.49	<0.001	1.43	1.25–1.64	<0.001
E-cigarette use intensity						
low use (<10 times/day) (ref.)	1.00	–	–	1.00	–	–
intensive use (≥10 times/day)	1.92	1.70–2.16	<0.001	–	–	–
Time to first use						
>30 min (ref.)	1.00	–	–	1.00	–	–
6–30 min		–	–	5.85	4.87–7.01	<0.001
≤5 min		–	–	19.08	15.90–22.90	<0.001
≤30 min	1.63	1.45–1.83	<0.001	–	–	–
Parental e-cigarette use						
no parental use (ref.)	1.00	–	–	1.00	–	–
any parental use	1.51	1.25–1.83	<0.001	1.70	1.39–2.07	<0.001
Parental traditional cigarette use						
neither parent (ref.)	1.00	–	–	1.00	–	–
1 parent	1.44	1.27–1.64	<0.001	–	–	–
mother only	–	–	–	1.67	1.37–2.04	<0.001
father only	–	–	–	1.85	1.57–2.18	<0.001
both parents	1.87	1.60–2.19	<0.001	2.13	1.79–2.54	<0.001
Parental awareness						
parents unaware (ref.)	1.00	–	–	–	–	–
parents aware	1.33	1.18–1.50	<0.001	1.65	1.45–1.87	<0.001
Risk perception						
perceived equally/more harmful (ref.)	1.00	–	–	1.00	–	–
perceived less harmful	1.40	1.24–1.58	<0.001	1.28	1.12–1.47	<0.001
Product initiation sequence						
e-cigarettes first (ref.)	1.00	–	–	–	–	–
traditional cigarettes first	1.89	1.67–2.13	<0.001	–	–	–
Primary motivation						
other reasons (ref.)	–	–	–	1.00	–	–
addiction/dependence	–	–	–	5.06	3.51–7.29	<0.001
smoking cessation	–	–	–	1.82	1.54–2.14	<0.001

“–” – The reference group or the absence of participants in the given category.

Male gender was associated with higher odds of both multiple product use (OR = 1.46, 95% CI: 1.30–1.64, p < 0.001) and intensive e-cigarette use (OR = 1.37, 95% CI: 1.21–1.56, p < 0.001). Age showed a linear relationship with both usage patterns, with each year increase associated with higher likelihood of multiple product use (OR = 1.18, 95% CI: 1.11–1.25, p < 0.001) and intensive e-cigarette use (OR = 1.22, 95% CI: 1.15–1.30, p < 0.001). Attending technical/vocational schools was associated with higher likelihood of both usage patterns compared to general high schools.

The intensity of e-cigarette use and multiple product use were strongly interconnected, with adolescents reporting intensive e-cigarette use nearly twice as likely to use multiple nicotine products (OR = 1.92, 95% CI: 1.70–2.16, p < 0.001). Time to first use after waking emerged as the strongest predictor of intensive e-cigarette use, with participants using e-cigarettes within 5 min of waking 19 times more likely to be intensive users compared to those waiting >30 min (OR = 19.08, 95% CI: 15.90–22.90, p < 0.001).

Use of e-cigarettes by either parent was associated with a 51% higher likelihood of multiple product use (OR = 1.51, 95% CI: 1.25–1.83, p < 0.001) and a 70% increase in the likelihood of intensive e-cigarette use (OR = 1.70, 95% CI: 1.39–2.07, p < 0.001). Traditional cigarette use by parents demonstrated a gradient effect: use by 1 parent increased the likelihood of multiple product use by 44% (OR = 1.44, 95% CI: 1.27–1.64, p < 0.001), while use by both parents elevated this likelihood by 87% (OR = 1.87, 95% CI: 1.60–2.19, p < 0.001). Perceiving e-cigarettes as less harmful than traditional cigarettes was associated with a 40% higher likelihood of multiple nicotine product use (OR = 1.40, 95% CI: 1.24–1.58, p < 0.001) and a 28% higher likelihood of intensive e-cigarette use (OR = 1.28, 95% CI: 1.12–1.47, p < 0.001). Nicotine initiation with traditional cigarettes (compared to e-cigarettes) was associated with almost twice the likelihood of using multiple nicotine products (OR = 1.89, 95% CI: 1.67–2.13, p < 0.001).

Self-reported addiction as the primary motivation for use was a strong predictor of intensive use, with these participants 5 times more likely to be intensive users compared to those reporting other motivations (OR = 5.06, 95% CI: 3.51–7.29, p < 0.001).

This analysis suggests a hierarchical structure of risk factors, with behavioral indicators of dependence showing the strongest associations, followed by family-related factors, cognitive factors, and demographic characteristics.

### Structural equation modeling of causal paths to e-cigarette use patterns

**Figure 1.**
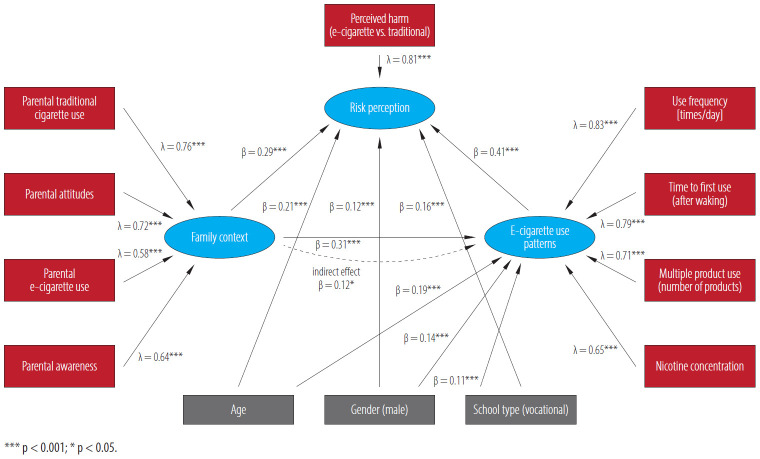
Structural equation model of family context, risk perception, and e-cigarette use patterns among Polish adolescents (N = 4797) aged 15–18 years, Poland, 2016–2020

Structural equation modeling examined the network of relationships between family context, risk perceptions, and e-cigarette use behaviors. The model demonstrated acceptable fit (χ^2^ = 142.67, df = 34, p < 0.001, CFI = 0.93, TLI = 0.91, RMSEA = 0.058, SRMR = 0.042) and revealed significant pathways (Table 5). A path diagram of the full model, including standardized path coefficients, is presented in Figure 1.

**Table 5.** Standardized path coefficients from structural equation model (N = 4797) – a cross-sectional study among 4797 Polish adolescents aged 15–18 years, Poland, 2016–2020

Path	β			p
	direct effect	indirect effect	total effect	
Family context				
e-cigarette use patterns	0.31	0.12	0.43	<0.001
risk perception	0.29	–	0.29	<0.001
Risk perception				
e-cigarette use patterns	0.41	–	0.41	<0.001
Age				
e-cigarette use patterns	0.19	0.08	0.27	<0.001
risk perception	0.21	–	0.21	<0.001
Gender (male)				
e-cigarette use patterns	0.14	0.06	0.20	<0.001
risk perception	0.16	–	0.16	<0.001
School type (vocational)				
e-cigarette use patterns	0.11	0.05	0.16	<0.001
risk perception	0.12	–	0.12	0.003

Family context had both direct (β = 0.31, p < 0.001) and indirect effects through risk perception (β = 0.12) on e-cigarette use patterns, with a substantial total effect (β = 0.43). Risk perception emerged as the strongest direct predictor (β = 0.41, p < 0.001). Age (total effect: β = 0.27), male gender (β = 0.20), and vocational school attendance (β = 0.16) all significantly predicted increased e-cigarette use.

Parental traditional cigarette use (λ = 0.76) and attitudes (λ = 0.72) were the strongest family context indicators, while perceived harm (λ = 0.81) was the primary risk perception indicator. For e-cigarette use patterns, use frequency (λ = 0.83) and time to first use (λ = 0.79) were the strongest indicators.

## DISCUSSION

This study provides comprehensive insights into the complex relationships between e-cigarette use patterns, family context, and risk perceptions among Polish adolescents. The authors' findings reveal alarming patterns of nicotine use, with 92.6% of adolescent e-cigarette users engaging in multiple product use and one-third reporting high-frequency use (≥10 times daily). The significant proportion using e-cigarettes within 5 min of waking (27.8%) suggests substantial nicotine dependence among young users.

Supporting the authors' hypothesis, adolescents who initiated with traditional cigarettes showed nearly twice the likelihood of multiple product use compared to e-cigarette initiators (OR = 1.89, 95% CI: 1.67–2.13). This finding aligns with Bold et al. [17] and contributes to a growing body of evidence on nicotine use progression pathways. While much attention has been focused on e-cigarettes as a potential “gateway” to smoking, the authors' data suggest the reverse pathway is also critically important: traditional cigarette initiation may act as a gateway to a more complex and entrenched pattern of multiple product use [17]. This may be because the rapid, high-dose nicotine delivery from combustible cigarettes establishes a stronger level of dependence, which in turn drives the user to seek out nicotine from multiple sources to maintain their desired levels throughout the day [18]. This public health concern is underscored by research measuring biomarkers of exposure, which shows that only smokers who completely switch to e-cigarettes significantly reduce their exposure to numerous key carcinogens and toxicants found in tobacco smoke. In contrast, dual users who continue to smoke fail to achieve these health benefits, remaining exposed to substantial levels of harmful constituents from combustible cigarettes [19].

Structural equation modeling demonstrated that family context exerts both direct (β = 0.31) and indirect effects through risk perceptions (β = 0.12) on adolescent e-cigarette use patterns. The combined effect (β = 0.43) underscores the family environment's substantial influence on adolescent nicotine behaviors. Notably, parental traditional cigarette use emerged as the strongest family context indicator (λ = 0.76), suggesting that parental smoking behaviors significantly shape adolescent e-cigarette adoption. This intergenerational transmission of smoking behavior is one of the most robust findings in tobacco control research, with large-scale meta-analyses confirming that parental smoking significantly increases the likelihood of smoking initiation among youth [20].

The hypothesis regarding parental e-cigarette use was confirmed, with parental use associated with 70% increased likelihood of intensive adolescent use (OR = 1.70, 95% CI: 1.39–2.07). This aligns with recent findings by Egger et al. [21], who documented higher vaping uptake among teenagers with parents who vaped or smoked.

Contrary to expectations, parental awareness was associated with higher, not lower, likelihood of intensive use. This finding likely reflects reverse causality – parents becoming aware only after problematic use patterns have developed, as suggested by Keenan et al. [22]. This interpretation is further supported by research demonstrating a significant discrepancy between adolescent self-reported substance use and parental awareness, with parents often underestimating or being unaware of their children's behavior, particularly in its early stages [23].

Similarly, the authors' findings regarding parental attitudes strongly supported the authors' hypothesis, with adolescents whose parents were supportive of e-cigarette use demonstrating the highest rates of high-frequency use (49.4%) compared to only 28.7% among adolescents whose parents opposed such use. These findings align with Trucco et al. [24], who demonstrated that parents' negative attitudes toward e-cigarettes were associated with weaker intentions to use among adolescents.

Moderation analyses revealed important developmental patterns. Family influence diminished with age (15–16 years: β = 0.38, 17–18 years: β = 0.25), while risk perceptions became more influential among older adolescents. This shift aligns with established developmental models wherein adolescence is characterized by a normative transformation of family dynamics; as peer influence grows, the parent-child relationship is renegotiated from a hierarchical structure to one of greater symmetry, reflecting the adolescent's increasing autonomy [25].

Risk perception emerged as the strongest direct predictor of e-cigarette use patterns (β = 0.41), a finding highly consistent with foundational health behavior theories, such as the health belief model, which posits that an individual's perception of risk is a primary determinant of their actions [26]. The authors' data show that 64.7% of participants perceived e-cigarettes as less harmful than traditional cigarettes. This misperception was associated with a 40% higher likelihood of multiple product use and a 28% higher likelihood of intensive use. This belief is not unique to Poland; international meta-analyses consistently show that a majority of adolescents view e-cigarettes as a safer alternative [27].

Gender differences in risk perception and use patterns revealed significant variations. Males demonstrated higher rates of both intensive use and multiple product use, reflecting global patterns where male adolescents consistently report higher tobacco use rates than females [28]. The authors' structural equation model further elucidated these pathways, showing that male gender was a significant predictor of both lower risk perception and, consequently, more intensive e-cigarette use patterns. This aligns with well-documented gender differences in risk-taking propensity and lower harm avoidance among male adolescents [29]. Additionally, self-reported addiction as a primary motivation was associated with a 5-fold higher likelihood of intensive use (OR = 5.06, 95% CI: 3.51–7.29), highlighting the importance of addressing both perceptions and motivations in prevention efforts. This demonstrates that subjective feelings of dependence are a robust indicator of compulsive use, aligning with core criteria for tobacco use disorder, such as loss of control over consumption [30].

The study's findings should be interpreted in light of its strengths and limitations. Key strengths include its large, nationally representative sample of adolescent e-cigarette users, providing valuable insights into the behaviors and perceptions of this specific high-risk subgroup. The application of structural equation modeling also allowed for a nuanced examination of the complex interplay between family factors, risk perceptions, and use patterns, moving beyond simple descriptive analysis.

Nevertheless, several limitations must be acknowledged. First, as the study design was cross-sectional, no causal relationships can be established, and the reliance on self-report data may be subject to recall or social desirability bias. Second, the generalizability of the findings is constrained by 2 key factors related to the study's scope: the data were collected in 2019, and the sample was, by design, limited to current e-cigarette users. Third, the low school-level response rate (5.5%) warrants caution, although stratification was used to mitigate this. Finally, the authors' SEM analysis treated ordinal indicator variables as continuous, which, while a common practice, is a simplification of the data structure.

Despite these limitations, the authors' findings highlight the need for comprehensive prevention strategies addressing both family and individual factors. The strong influence of parental behaviors and attitudes suggests that family-based interventions could be particularly effective, while correcting misperceptions about e-cigarette harm appears crucial, especially among male adolescents who show stronger risk perception-behavior associations.

## CONCLUSIONS

In conclusion, this study reveals the complex interplay of behavioral, family, and cognitive factors driving adolescent nicotine use in Poland. The alarmingly high prevalence of multiple product use, fueled by strong parental influences and low-risk perceptions, calls for an urgent, multi-faceted public health response.

The findings from this research have several direct implications. Primarily, the discovery that >90% of adolescent vapers use multiple nicotine products is a clear signal that policies and interventions focused solely on e-cigarettes are insufficient. To be effective, prevention campaigns and clinical screening must adopt a comprehensive approach that addresses the entire ecosystem of nicotine products available to youth. Given the strong influence of family context, interventions should target parents as critical agents of change. Public health programs should aim to educate parents on the powerful impact of their own smoking and vaping behaviors, and clinicians, such as pediatricians, are uniquely positioned to provide this counseling. The authors' findings also support a tailored approach to prevention. For younger adolescents, interventions should prioritize strengthening family protective factors, while for older adolescents, where individual cognitions become more dominant, programs should focus on correcting risk misperceptions.

Future research should build on these cross-sectional findings. Longitudinal studies are needed to confirm the causal pathways suggested by the authors' modeling, tracking the developmental transitions between parental influence, risk perception, and adolescent behavior over time. Finally, there is a pressing need for intervention research to design and evaluate the effectiveness of the family-based and tailored communication strategies proposed here.
